# Simultaneously Characterizing the Volatility Distribution and Phase State of Submicron Secondary Organic Aerosols Using a Vocus Vaporization Inlet for Aerosols with a Chemical Ionization Mass Spectrometer

**DOI:** 10.1021/acsestair.5c00155

**Published:** 2025-10-15

**Authors:** Sining Niu, Kyle P. McCary, Mitchell Alton, Jordan E. Krechmer, Harald Stark, Jason D. Surratt, Manjula Canagaratna, Yue Zhang

**Affiliations:** † Department of Atmospheric Sciences, 14736 Texas A&M University, College Station, Texas 77843, United States; ‡ Center for Aerosol & Cloud Chemistry, 53777 Aerodyne Research, Billerica, Massachusetts 01821, United States; § Department of Chemistry, University of Colorado Boulder, and Cooperative Institute for Research in Environmental Sciences, Boulder, Colorado 80309, United States; ∥ Department of Environmental Sciences and Engineering, 2331 The University of North Carolina at Chapel Hill, Chapel Hill, North Carolina 27599, United States; ⊥ Department of Chemistry, The University of North Carolina at Chapel Hill, Chapel Hill, North Carolina 27599, United States

**Keywords:** volatility basis set, glass transition temperature, viscosity, secondary organic aerosols (SOA), ammonium mode chemical ionization mass spectrometer

## Abstract

Volatility and viscosity are important parameters affecting the formation, reaction, and fate of atmospheric organic aerosols. In this study, a Vaporization Inlet for Aerosol (VIA) coupled with a Vocus chemical ionization mass spectrometer (Vocus-CIMS) using NH_4_^+^ adduct ionization is employed to simultaneously detect and quantify the molecular composition and volatility of organic aerosols through a program-controlled temperature ramp, thereby providing viscosity information. Volatility calibration was conducted with a series of reference aerosol particles with different chemical compositions, covering a vapor pressure range from 10^–1^ to 10^–8^ Pa. Secondary organic aerosols (SOA) produced from the potential aerosol mass reactor were analyzed by the VIA-CIMS. Chemical species ranging from semivolatile to low-volatility, including highly oxygenated dimers, were identified. Individual ions from the collected mass spectra were fitted and grouped by volatility basis sets to yield the volatility distribution of the SOA, allowing for the quantification of the glass transition temperatures and viscosities. Results show that β-caryophyllene ozonolysis SOA has lower volatility and is more viscous than the α-pinene SOA. This approach enables the online quantification of SOA particle chemical composition and volatility distribution, while simultaneously characterizing particle phase state, such as viscosity and water diffusion time, providing crucial insights into their chemical processes and climate impacts.

## Graphical abstract

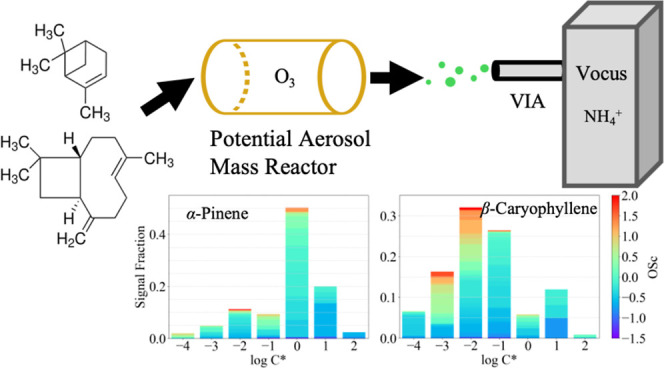

## 1 Introduction

Organic aerosols (OAs) are ubiquitous in the atmosphere and play critical roles in air quality and climate forcing.^1,2^ Secondary organic aerosols (SOA) constitute a significant fraction of submicron OA in remote regions such as the Amazon forest^3–5^ and make up to 20–80% of measured particulate matter mass depending on seasonal and regional variability.^6–11^ SOA is formed through the oxidation of volatile organic compounds (VOCs)^2,12^ and has been shown to affect climate directly by absorbing and scattering light^13–15^ and indirectly by affecting the number concentration of cloud condensation nuclei^16–18^ and ice nucleating particles.^19^

However, the chemical reaction kinetics and the climate effects of SOA are not yet fully understood, partially due to limited knowledge of SOA volatility and viscosity (η).^20^ Volatility influences the gas-particle partitioning of organic compounds,^21^ thereby affecting SOA formation and growth. For instance, low-volatility organic compounds tend to either nucleate or condense onto existing particles, contributing to particle mass growth while influencing the optical properties and atmospheric lifetime of aerosols.^22^ Viscosity, on the other hand, affects the diffusion rates of molecules within particles, which in turn affects particle growth, multiphase chemical reactions,^23^ and mixing state.^24^ Moreover, viscosity affects the SOA phase state and ability to act as ice-nucleating particles.^19^ For instance, when in a glassy or semisolid phase, SOA may act as effective heterogeneous ice nuclei.^19,25–30^ Despite being important, the volatility and viscosity of SOA are often not well characterized,^31,32^ leading to uncertainties in predicting aerosol properties in chemical transportation and climate models.^33^

Different methods have been utilized to improve the characterizations of SOA volatility, including in situ measurements and modeling. Thermal desorption coupled with mass spectrometry has been utilized to measure the volatility distribution of SOA in both laboratory experiments and field studies.^34–40^ For example, an aerosol mass spectrometer (AMS) coupled with a thermal denuder is used^41–44^ due to its reproducibility and linear superposition of mass spectra.^45,46^ However, significant thermal decomposition and fragmentation caused by the high-temperature vaporizer and electron impact ionization^45^ prevent direct identification of chemical molecules, often leading to potential overestimation of volatility and underestimation of viscosity.^47^ In addition, online chemical ionization mass spectrometry (CIMS), a softer ionization technique, coupled with the Filter Inlet for Gases and AEROsols (FIGAERO), has been developed to detect the chemical composition and volatility distribution of atmospheric aerosols with less fragmentation.^48,49^ The usage of FIGAERO allows for simultaneous measurements of volatility and chemical composition, as aerosol particles are first collected on a Teflon filter and then thermally desorbed via temperature-programmed heating for mass spectrometric analysis.^50,51^ However, absorption of semivolatile organic compounds onto the Teflon filter during the sample collection can introduce artifacts in volatility measurements.^52,53^ Several alternative thermogram-based techniques have also been developed, including ChemSpot^54^ and volatility and polarity separator (VAPS).^55^ ChemSpot utilizes stepped thermal desorption coupled with flame ionization detection to generate volatility thermograms. While ChemSpot provides a simple and cost-effective approach for routine volatility monitoring, it lacks molecular-level resolution.^54^ The VAPS utilizes a parallel heating and derivatization system to separate OA based on both volatility and polarity. Several studies have compared these approaches with more compositionally resolved techniques, and they highlighted significant variability in volatility estimation across methods and the need for techniques that offer both volatility estimation and molecular-level information. In addition, heating experiments inside an environmental transmission electron microscope (ETEM) can provide information regarding the volatility of different species but are limited by missing high-volatility species in a high-vacuum ETEM chamber in addition to the small number of particles analyzed.^56^ Other than thermal measurements, an evaporation chamber under room temperature has also been employed to characterize the aerosol volatility.^57,58^ In addition to the direct measurements, volatility can also be derived from formula-based group contribution methods.^59,60^ The formula-based methods allow for rapid volatility prediction based on molecular composition only and are useful for large-scale modeling. However, they are constrained by reliance on empirical parametrization and limited experimental data sets. Therefore, these methods may misestimate the volatility of highly oxygenated species, particularly in complex mixtures.^61^

Compared with volatility measurements, quantifying the viscosity of aerosol particles is often more challenging.^62^ Several existing methods, including the evaluation of morphology change from individual particles,^63–65^ optical tweezers,^66–69^ bead mobility,^70^ and tilted scanning electron microscopy, are used to quantify the viscosity of organic particles.^71,72^ However, some online measurement techniques, such as optical tweezers, for example, require milligrams of sample for atomization, and such a mass is difficult to obtain from ambient aerosol particles.^62^ Other online measurements, including the morphology change and particle rebound, often need delicate controlled experimental conditions and do not provide information related to chemical composition.^73,74^

Recently, various models have also been developed to predict the volatility and viscosity of pure compounds and mixtures based on their chemical composition measured by offline high-resolution mass spectrometry.^68,75–78^ However, due to the rapid fluctuations in the concentrations and compositions of ambient SOA, there is a pressing need to provide near real-time quantification of the viscosity, volatility, and molecular composition of submicron SOA systems.^79^

To address the above knowledge gap, a thermal desorption unit coupled with CIMS, together with semiempirical modeling,^80^ was employed to quantify the volatility distribution, glass transition temperature, and viscosity of laboratory-generated SOA through ozonolysis of α-pinene and β-caryophyllene. This method includes the state-of-art Vaporization Inlet for Aerosols (VIA, Aerodyne Research, Inc.) coupled with NH_4_^+^ Vocus-CIMS, providing broader detection of oxygenated ions and distinct ionization characteristics compared to the nitrate CIMS used in previous work,^81^ while thermally evaporating and detecting the chemical composition of the aerosols. Compared with FIGAERO, this technique avoids the sample collection and enables measurements with high time resolution.^81,82^ Thermal decomposition is also reduced due to the shorter residence time (0.1 s with 3 L per minute flow rate) of the aerosol particles in the VIA.^81^ In this study, the full volatility basis set distribution was derived from the VIA thermogram upon volatility calibration. The glass transition temperatures were then determined to be 268 and 290 K for α-pinene and β-caryophyllene SOA, respectively. This study assesses the viscosity of SOA mixtures through a bottom-up approach, incorporating their chemical composition and mass fraction. Notably, it demonstrated that even at room temperature β-caryophyllene SOA can remain in the glassy phase under low relative humidity (RH) conditions.

## 2 Materials and Methods

### 2.1 Operation of Vocus VIA

#### 2.1.1 Working Principle of the NH_4_^+^ Vocus

The Vocus-CIMS was utilized in this study with ammonium-water clusters (NH_4_^+^·H_2_O) as the reagent ion, which is able to detect a wide range of oxygenated organic compounds and leads to less fragmentation compared with proton transfer reaction MS.^83^ Detailed descriptions of the NH_4_^+^ Vocus were discussed in previous studies.^83–85^ Briefly, the Vocus consisted of an ion source, a focusing ion–molecule reactor (FIMR), and a long time-of-flight mass spectrometer with a 1.5 m-long flight tube.^84^ The NH_4_^+^·H_2_O was generated in the ion source with the vapor from a 0.5 vol % ammonium hydroxide water solution. The water molecule was more abundant in the system, and therefore, H_3_O^+^ was the primary ion produced by the plasma formed between the conical surfaces. The proton transfer reaction between NH_3_ and H_3_O^+^ then produced NH_4_^+^, as illustrated in R1. Subsequently, NH_4_^+^ ions clustered with excess water molecules and NH_3_, as shown in R2 and R3. Other than the ammonium cluster, H_3_O^+^ could also cluster with water molecules, as shown in R4. Overall, several ion clusters were produced in the system and served as reagent ions.

R1 $${H_{3}O}^{+}+{NH}_{3}\to {NH}_{4}^{+}+H_{2}O$$

R2 $${NH}_{4}^{+}+{nH}_{2}O\to {NH}_{4}^{+}\cdot {\left(H_{2}O\right)}_{n}$$

$${NH}_{4}^{+}+{nNH}_{3}\to {NH}_{4}^{+}\cdot {\left({NH}_{3}\right)}_{n}$$ R3

$${H_{3}O}^{+}+{nH}_{2}O\to {H_{3}O}^{+}\cdot {\left(H_{2}O\right)}_{n}$$ R4

The reagent ions then entered the FIMR, comprised of a 100 mm long and 10 mm wide (inner diameter) glass tube inside of a quadrupole radio frequency field, to ionize the 100 standard cubic centimeters per minute (sccm) sample flow. The ions exiting FIMR passed through a big segmented quadrupole to partially filter out the massive signal from low mass reagent ions.^83^ The ions were then guided to a long time-of-flight mass analyzer that achieved a mass resolving power (*m*/Δ*m*, defined at full width at half-maximum) up to 10,000. The Vocus was calibrated periodically with a multicomponent VOC standard from a prepared cylinder (Apel-Riemer Environmental) to correct any instrumental instability during the experiments. All of the Vocus data were analyzed with the Aerodyne package Tofware 4.0.0 in Igor Pro (WaveMetrics Inc., Version 9).

#### 2.1.2 Operation Procedures for the Vaporization Inlet for Aerosols

The VIA was an inlet designed for particle evaporation, providing online measurements of compounds in the particle phase when coupled with gas monitors for chemical analysis. Detailed descriptions and characterizations of the VIA were discussed in previous literature.^81,82^ Briefly, the VIA consisted of a gas denuder and a thermal vaporization unit. The activated charcoal denuder removed the preexisting gas phase compounds in the sampling air so that the remaining particles evaporated in the thermal desorption unit. The thermal desorption unit consisted of a 40 cm long Sulfinert-coated stainless steel tubing with a 1 in. outer diameter. The total flow rate passing through the VIA was 3 L/min (LPM), corresponding to a residence time of 0.1 s. After exiting the thermal desorption unit, around 100 sccm of sample air entered the Vocus for chemical analysis, while the excess air was drawn to a vacuum pump as exhaust. The charcoal denuder was flushed with heated air after each experiment for regeneration. The VIA was heated from 25 to 230 °C, as measured using a Pt100 sensor on the outside surface of the oven. A controlled temperature ramp was performed on VIA to linearly increase the temperature. The proportional–integral–derivative controller was tuned for the best linear temperature ramping with *k*_p_ = 60, *k*_i_ = 2, and *k*_d_ = 1, as shown in Figure S1.

### 2.2 Experimental Setup

#### 2.2.1 Organic Standards and SOA Generation

A series of laboratory experiments consisting of OA standards and SOA were conducted with the experimental setup shown in Figure 1. A constant output atomizer (TSI, model 3076) was used to produce the OA of individual standards from their aqueous solution. In addition, SOA were generated from the ozonolysis products of selected VOC precursors in a potential aerosol mass (PAM) oxidation flow reactor (Aerodyne Research, Inc.) under dark and dry conditions with a RH of <3%.^86,87^ Briefly, the VOC precursors were each injected into a three-necked manifold by a syringe pump (Chemyx, Model Fusion 400) and carried out by a 3 LPM flow of zero air from a zero air generator (AADCO, model 737-14-A-CH4–120). Ozone was generated through an in-house customized ozone generator at a flow rate of 3 LPM.^88^ The above flow rate settings led to a concentration of VOCs at 100 ppb, and the homogeneously nucleated SOA particles were sampled by VIA-CIMS downstream. The particle volume and size distributions were stable across all the experiments; therefore, the effects of size on thermal desorption temperature were minimum in this study. However, future work should examine the dependence of VIA performance on particle size, particularly for ambient sampling, which often has variable size distributions.

**Figure 1:**
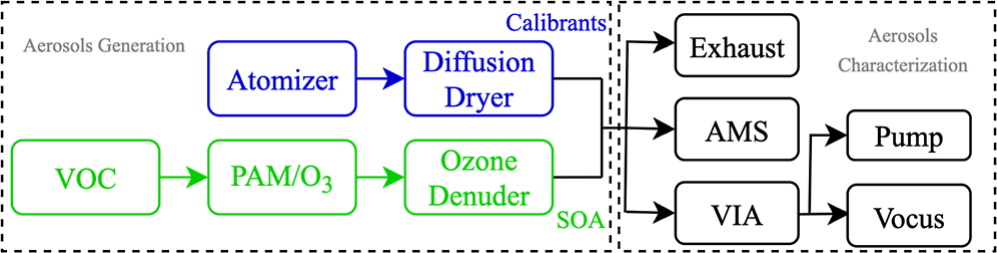
A schematic diagram of the experimental setup.

#### 2.2.2 Operation Procedures of the Aerosol Mass Spectrometer

A high-resolution time-of-flight AMS (HR-ToF-AMS, herein referred to as AMS) was deployed in the experiments for quantitative measurements of OA. The detailed working principles of the AMS were discussed in previous studies.^45,46,89,90^ Briefly, the aerosol particles are focused through the aerodynamic lens and enter the vacuum as a narrow beam. The particles are then directed to a vaporizer, followed by an electron impact ionizer. The ionized molecules from nonrefractory aerosol particles will then enter a ToF mass spectrometer. The high mass resolution (up to 4000) enables the differentiation of ions with the same nominal mass. The AMS was calibrated with ammonium nitrate before experiments, with the calibration method described previously.^45,46^ Though the AMS usually has a lower size limit at around 50 nm,^91^ as shown in Figure S2, particles smaller than 50 nm only contribute to less than 2% of the total aerosol volume, making the discrepancy in the particle volume measured by AMS and Vocus negligible. Therefore, the AMS was used to quantify the mass concentration of OA as well as the residual mass after the VIA.^45,46,89,90^ The signals from Vocus were corrected with the mass loading measured by the AMS to account for any minor instability of the aerosol generation segment by normalizing the measured mass concentration to the initial mass concentration. Additionally, the AMS was directly connected after VIA to quantify the evaporation efficiency of VIA at the maximum temperature. The AMS was deployed in V mode, and the data were analyzed with the Squirrel (version 1.65) and Pika (version 1.25) packages in Igor Pro (WaveMetrics Inc., version 8).

### 2.3 Thermogram Method

#### 2.3.1 VIA Volatility Calibration

The volatility of different species was estimated from their thermal desorption temperature using a calibration curve established with carefully selected calibrants whose volatility was known from previous studies, as shown in Table S1. The thermograms of calibrants were obtained with Vocus by ramping the VIA temperature as the setting described in Section 2.1.2. Multiple carboxylic acids, including *cis*-pinonic acid (Sigma-Aldrich, 98% purity), glutaric acid (Sigma-Aldrich, 99% purity), adipic acid (Sigma-Aldrich, 99% purity), azelaic acid (Sigma-Aldrich, 98% purity), sebacic acid (Sigma-Aldrich, 99% purity), and dodecanedioic acid (Sigma-Aldrich, 99% purity), as well as polyethylene glycol-8 (PEG-8, Sigma-Aldrich, 95% purity), were selected as calibrants. These calibrants covered a wide volatility range of saturation mass concentrations (*C*_0_), with a common logarithm of the *C*_0_ (log *C*_0_) values ranging from −3 to 3. The effective saturation concentration (C*), which considered the nonideality of mixing of different aerosol components, was derived from thermograms of complex SOA mixtures. With the approximation C* ≈ *C*_0_, the C* values were applied in equations originally formulated for *C*_0_. Despite the above simplification, this approach allowed for consistent application of semiempirical models and facilitated comparison with previous studies.^92^

To ensure that the signals detected through VIA Vocus were from aerosol particles instead of the background from the instrument, blank measurements were conducted before each calibrant. A particle filter was placed at the VIA inlet to remove any particles in zero air during the blank experiments. Blank measurements were performed with the same VIA setting as those for the other measurements. The thermograms for all the calibrants are shown in Figure S3. A constant aerosol flow of calibrants was introduced into the VIA, and their signals reached a plateau after fully evaporation.^81^ After background subtraction and calibration were performed before and after the ramp, all calibrant thermograms exhibited a sigmoid shape. These thermograms were subsequently fitted using a sigmoid function, as shown in equation (eq 1):

1 $$Signal=\frac{a}{1+\exp\left(-\frac{T-T_{50}}{b}\right)}$$

where *a* is the maximum value of the fitted function and *b* is the steepness of the curve. The *T*_50_ is defined as the temperature at which half of the signal is obtained, which is the midpoint of the function. With the establishment of the calibration curve (details in Section 3.1), the C* of the target compounds was derived with their measured *T*_50_. Despite that most of the thermograms showed sigmoid-curve shapes, a couple of thermograms, including those from glutaric and pinonic acid, showed a smaller peak, as shown in Figure S3. Such peak shapes may partially be attributed to thermal readsorption. While this behavior was not observed for other calibrants, it is consistent with previous studies in thermal desorption systems,^81^ and further targeted studies would be helpful to investigate the mechanism.

Other than the C*, the evaporation enthalpy (Δ*H*) for individual species was derived from the linear fitting of the signal intensity plotted versus 1/*T* with the Clausius–Clapeyron equation (eq 2) below:^39,93^

2 $$\ln\left(I_{T}-I_{T_{0}}\right)=-\frac{\Delta H}{RT}+Const$$

where *I*_*T*_ and *I*_*T*0_ are the signal intensities at VIA temperature *T* and at room temperature *T*_0_, respectively.

#### 2.3.2 Identification of the Thermal Decomposition Products of SOA

Similar to the calibration experiments, a blank experiment was conducted before each SOA experiment. The thermal decomposition of SOA was also considered and corrected after initial HR analysis, calibration, and background subtraction. To locate the thermally decomposed product ions, the sigmoid function was used to fit the thermogram of each HR compound. The *T*_50_ retrieved from the fitting was converted to a corresponding measured C* (*C*_m_*) from the calibration curve established from the VIA volatility calibration experiments. In addition to the measurements, the predicted C* (*C*_p_*) of each organic compound was also derived from a previously established model that uses oxygen and carbon numbers to estimate volatility.^60^ Detailed information on the model was described in a previous study.^60^ By comparing the C* values, if the *C*_m_* was greater than 10 × *C*_p_*, we consider the compound as a thermal decomposition product ion, as indicated by its lower volatility in the thermogram measurements compared to model predictions. This 10-time threshold was supported by earlier findings that the volatility model prediction method used here tends to underestimate true volatility;^61^ therefore, the actual volatility cutoff to determine thermal decomposition products may be several orders of magnitude lower than the *C*_m_*.^94^ To further test the sensitivity of the cutoff threshold and the robustness of such a cutoff, we performed the analysis using the 5 and 100 time cutoff. As shown in Table S4, the resulting volatility distribution and *T*_g_ remained unchanged. The sensitivity analysis suggested that the results were robust against the 10× cutoff threshold. With this criterion, around 20 to 30% of the detected ions were potential decomposition products. This number was likely an overestimated decomposition fraction, as a single parent molecule can produce multiple product ions that artificially increase the percentage of decomposition products.

## 3 Results and Discussion

### 3.1 Calibration Curve

With the selected calibrants and using the thermogram method, a calibration curve of VIA NH_4_^+^ Vocus was established for volatility. As shown in Figure 2, the whole temperature range calibration was achieved by linear regression of individual *T*_50_ and logC* for all of the calibrants. The *T*_50_ was the average measured thermal desorption temperature, and C* was derived by applying the saturation vapor pressure (*p*_v_) measured through the thermal desorption method from literature into eq 3:^79,95^

3 $$C^{*}=\frac{p_{v}\times MW}{R\times 298K}$$

where MW is the molecular weight of each calibrant compound and *R* is the universal gas constant.

**Figure 2:**
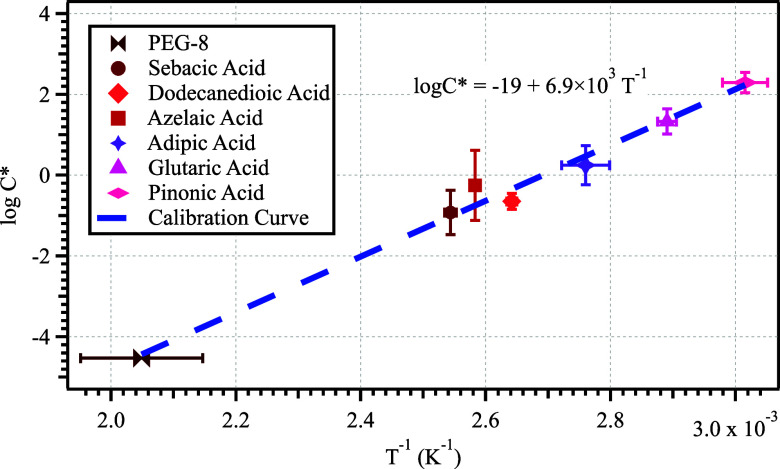
VIA volatility calibration curve (*R* ^2^ = 0.99). The *y*-axis is the reported C* of the calibrants, and the *x*-axis is the inverse of the corresponding thermal desorption temperature from VIA NH_4_+ Vocus measurements. Uncertainty range of each data point on the *x*-axis is derived from three measurements and that on the *y*-axis is from reported literature values.

As shown in the calibration plot (Figure 2), the uncertainties mostly come from the variability of the vapor pressure values (*p*_v_) derived from the literature. The uncertainties associated with the thermogram fitting were generally small, showing high reproducibility of the measurements. The logC* values obtained from the literature and those derived from the measured Δ*H* based on West et al.^39^ demonstrated a linear correlation with *R* ^2^ = 0.85 and a slope of 1.6, as shown in Figure S4. Using the slope of the regression curve shown in Figure S4, the logC* derived from the evaporation enthalpy method discussed in this study was corrected to allow for a direct comparison.

### 3.2 Chemical Composition of SOA Obtained by Vocus VIA

By applying the data processing methods described in Section 2.3.2, the full chemical formula lists were obtained for α-pinene and β-caryophyllene SOA. Due to the lack of authentic standards, all the signal intensities were represented as counts per second (cps) without deriving actual concentrations. To characterize the chemical composition of the particle-phase organic species, the mass spectra obtained at the maximum temperature (220 °C) were selected, as most of the compounds had fully desorbed at this temperature. Potential thermal decomposition products were excluded from the analysis as mentioned in Section 2.3.2. The detected organic compounds were dominated by the CHO group and mainly divided into two classes: monomers and dimers, based on their carbon number.

The total signal of α-pinene SOA was dominated by monomers with 5–10 carbons. C_10_H_16_O_2_, C_8_H_12_O_4_, C_9_H_14_O_4_, C_10_H_16_O_4_, and C_10_H_16_O_6_ were the major components for monomers, as shown in Figures 3 and S5. The dimers, consisting of compounds with 15–20 carbons, accounted for only ∼5% of the total signal compared to the monomers but remained clearly distinguishable from the baseline. Dimers were dominated by C_18_H_30_O_9_, C_19_H_28_O_7_, C_19_H_30_O_6_, C_16_H_24_O_5_, and C_18_H_28_O_8_.

**Figure 3:**
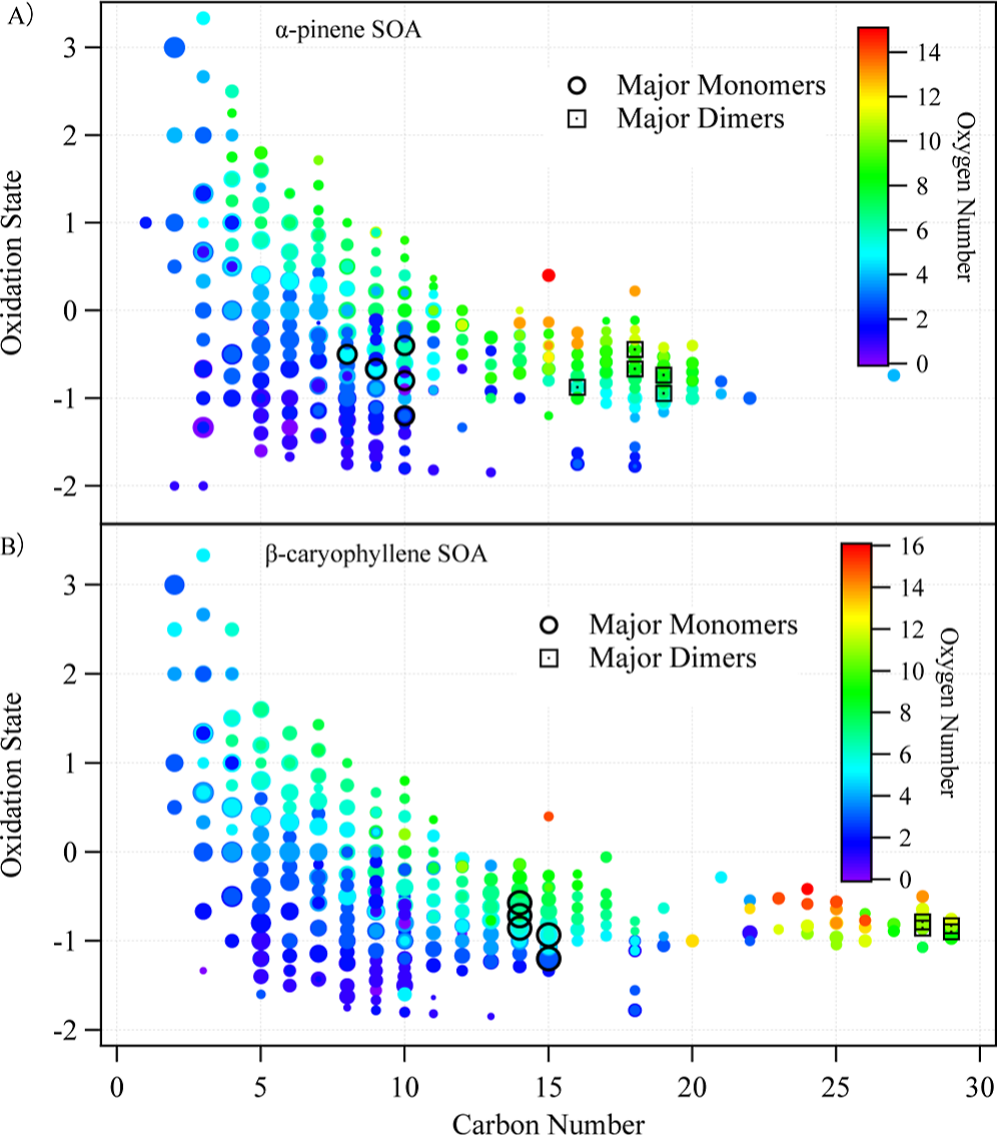
Carbon number and oxidation state of each SOA species measured with VIA NH_4_+ Vocus. The major monomers and dimers were denoted with circles and squared dots, respectively. The chemical formulas of the monomers and dimers are described in text. The size of each circle and square is proportional to the logarithm of the signal intensity.

Similar to α-pinene SOA, the signal of β-caryophyllene SOA was also dominated by monomers, including C_14_H_22_O_6_, C_15_H_24_O_3_, C_14_H_22_O_7_, C_14_H_22_O_5_, and C_15_H_24_O_5_. The dimers were also detected, with C_28_H_44_O_11_, C_28_H_42_O_10_, C_29_H_44_O_10_, C_28_H_44_O_10_, and C_29_H_44_O_9_ being the most significant ions. The majority of dominating signals of monomers and dimers identified in previous measurements were detected by the VIA Vocus,^82,96–98^ suggesting the chemical composition of the SOA, especially oligomeric compounds, was similar to SOA produced from previous studies, allowing for direct viscosity comparison.^3,97,99^ The Kendrick mass defect plots for both SOA are shown in Figure S5, showing chemical series related by the addition of organic compound groups such as –CH_2_ and –O. The mass defect plot visualized the elemental composition patterns of complex SOA mixtures and enabled the examination of multiple groups of compounds.^100^ It is worth noting that the trimers were not detected in this study, potentially due to the selectivity of the reagent ions^101^ and the temperature range of VIA used. The trimers often accounted for a small portion of the total SOA mass (∼5–10%)^82,102^ and were estimated not to affect the overall general volatility distribution of the SOA within the uncertainty range.

Table S2 shows the O/C ratio and average oxidation state of the carbon ( $\overset{®}{{OS}_{C}}$ )^103^ derived from both the AMS and VIA Vocus, with the latter assuming uniform ionization efficiency across all species. The average chemical formula was derived from VIA Vocus with the relative intensity for each compound as the weight. In general, β-caryophyllene SOA showed a longer average carbon chain than α-pinene SOA, consistent with their original carbon skeleton. In addition, the β-caryophyllene SOA has a slightly lower oxidation state.

### 3.3 Volatility Distribution of SOA

#### 3.3.1 Volatility Calculation Using the Thermogram of Total Ion Counts

As discussed in Section 2.3, the processed total thermogram of SOA measured from VIA Vocus was obtained by increasing the temperature of the VIA from a stable source of SOA generated by the PAM reactor. Figure S6 illustrates the measured total thermogram signal and the fitting curves for both SOA types based on the raw signal without background subtraction. Using the volatility basis set, seven sigmoid functions were combined to fit the total thermograms (i.e., predetermined logC* values, with predefined logC* values ranging from −4 to 2). Despite the short residence time in the VIA, some highly volatile compounds were detected at room temperature. Therefore, the intermediate VOCs and semi-VOCs products with apparent saturation concentrations larger than 100 μg/m^3^ were assigned to the most volatile bin (logC* = 2) due to the limitation of room temperature measurements. However, such an assignment might slightly underestimate the overall volatility distribution due to the omission of more volatile components (logC* >2).

To overcome such an issue, the low-temperature baseline visible in Figure S6 was removed through background subtraction to generate Figure 4 as discussed in Section 3.3.2. The signal intensity for each volatility bin was derived from the amplitude of the corresponding fitted sigmoid functions. The sum of the fittings for all the volatility bins agreed well with the measurements in both SOA, with *R* ^2^ = 0.99, demonstrating the accurate allocation of the volatility distribution.

**Figure 4:**
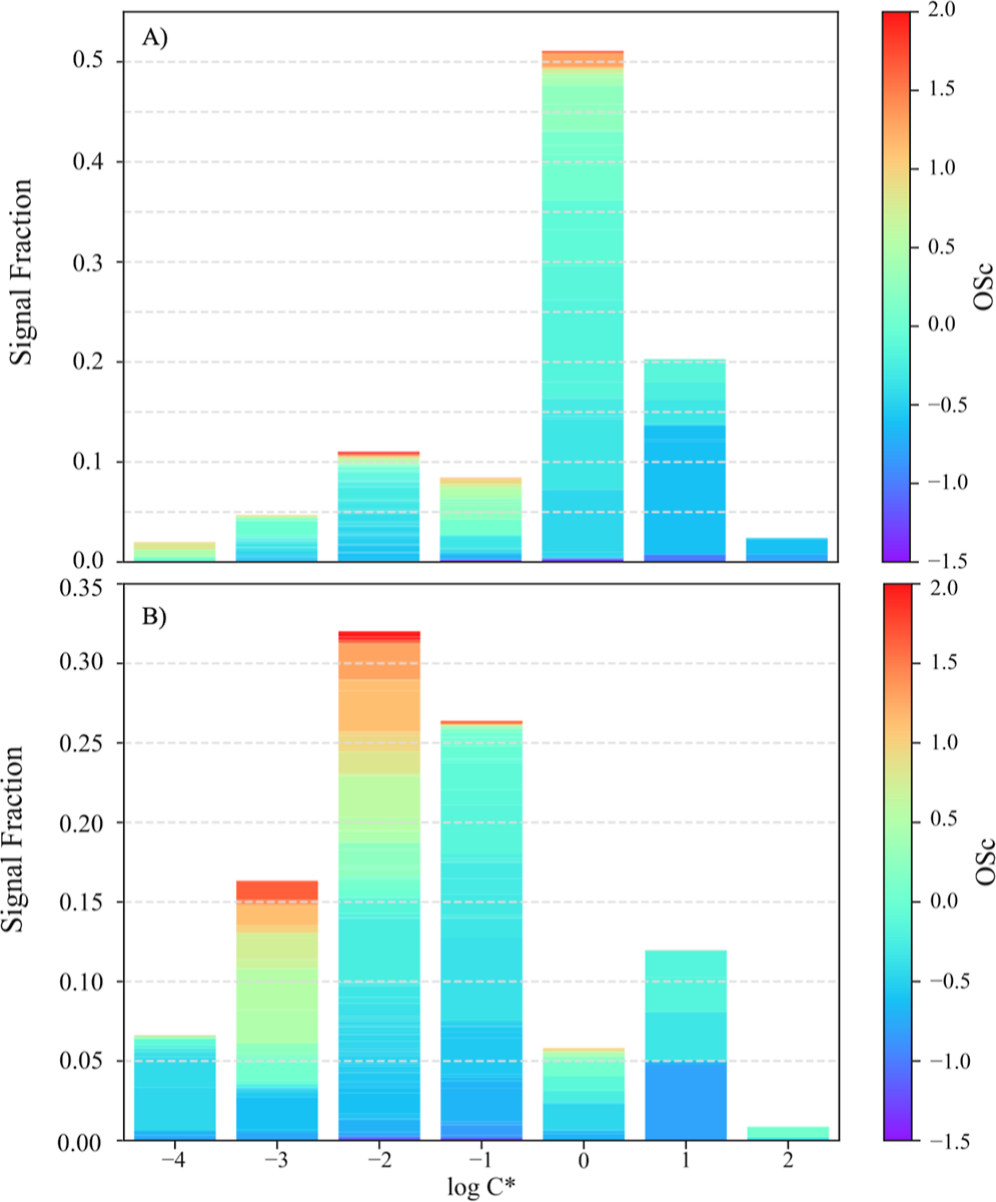
Volatility basis set derived from individual thermogram fitting, color-coded with different oxidation state ranges for (A) α-pinene and (B) β-caryophyllene SOA.

#### 3.3.2 Volatility Calculation Using Each Individual Thermogram

Given the high mass resolution of Vocus, the chemical formulas of most of the ions were successfully identified, allowing for plotting the thermograms for individual signals, as shown in Figure S7. The thermogram of each detected signal was fitted by a sigmoid function. The volatility information was derived from the corresponding fitting results *T*_50_ with the calibration curve established in Section 3.1. Within the same SOA mixture, the volatility information for a series of selected organic compounds is shown in Figure S7. For α-pinene SOA group C_10_H_16_O_3–7_, their thermal desorption temperature and volatility are positively and negatively correlated to the *T*_50_ of the individual ion, respectively. This trend is consistent for the β-caryophyllene SOA group C_15_H_22_O_3–6_. The slight inversion observed between the *T*_50_ values of C_15_H_22_O_6_ and C_15_H_22_O_5_ is likely due to the proximity of their thermogram. The marginally higher *T*_50_ of C_15_H_22_O_5_ of 3 K is not considered significant, as the difference is within the uncertainty of *T*_50_ determination. It is worth noting that each molecular formula detected by Vocus-CIMS might represent multiple isomers, and they were not distinguished in this study. Therefore, the observed thermogram for a signal may represent a mixture of species, and structural ambiguity remains a source of uncertainty in interpreting volatility.

With the derived C*, all detected signals were clustered based on their logC* for a clearer illustration of the volatility distribution of the SOA particle phase. As shown in Figure 4, α-pinene SOA was more volatile compared with β-caryophyllene SOA, with a shorter average carbon chain and a lower average oxidation state, as discussed in Section 3.3.2. A general trend observed here is that compounds with lower volatility tend to exhibit a higher $\overset{®}{{OS}_{C}}$ and O/C ratio, consistent with previous observations.^77,104^ The relatively lower $\overset{®}{{OS}_{C}}$ for β-caryophyllene SOA with logC* = −4 was attributed to oligomers with long carbon chains, which were formed through nonoxidative reactions, consistent with previous studies.^104^ The volatility distribution of α-pinene SOA demonstrated here agreed with previous studies utilizing TDMS and SOA mass yield fittings, both of which showed volatility peaked at logC* = 0.^104,105^ Previous studies investigating β-caryophyllene SOA reported a more volatile VBS distribution,^96,106^ likely reflecting methodological differences. Tasoglou et al. inferred volatility from SOA yield and a four product VBS framework,^106^ while Gao et al. applied FIGAERO–CIMS with I^–^ as the adduct ion for positive matrix factorization to derive the volatility distribution.^96^ The different ionization selectivity and the inherent modeling assumption likely contribute to the observed differences. Notably, a previous study utilizing a TD scanning mobility particle spectrometer showed similar results compared with this study, with a peak of VBS at logC* = −1.^107^ In general, the volatility distribution of SOA varied across previous studies due to differences in measurement techniques.^61^

As discussed in Section 3.1, the evaporation enthalpy of each detected signal can be calculated using its respective thermogram based on the work from West et al.^39^ The derived logC* values from evaporation enthalpy were corrected using the linear regression from Figure S4 and plotted versus those derived from *T*_50_, as shown in Figure S8. The linear correlation between the derived logC* from two methods, with a slope of 1.1 and a small intercept of −0.15, demonstrates the ability to predict the volatility of organic compounds using the thermogram and Δ*H*.

### 3.4 The Glass Transition Temperature and Viscosity of SOA

Previous studies show that the vapor pressure and viscosity are both influenced by the van der Waals forces between the molecules.^108^ A stronger van der Waals force leads to a lower volatility and higher glass transition temperature (*T*_g_), which positively correlates to the viscosity.^108^ Therefore, the *T*_g_ can be calculated from C* using a semiempirical relation developed in previous studies, as shown in eq 4.^76,80^

4 $$T_{g,i}=480.07-\frac{54395}{{\left(\log\left(\frac{RT}{\xi ΜW}C_{i}^{*}\right)-7.7929\right)}^{2}+116.49}$$

where *T* (in K) represents the reaction temperature in PAM, set at 298 K, R is the ideal gas law constant, MW is the molecular weight of organic compound *i*, *C*_i_* is the C* of organic compound *i* (in μg/m^3^), and ξ is the activity coefficient, which was assumed to be unity. To further derive the *T*_g_ for the SOA mixture under dry conditions, the Gordon–Taylor equation^109^ was implemented by assuming the Gordon–Taylor constant of 1, given that these are organic mixtures,^110^ as shown in eq 5:

5 $$T_{g,org}=\sum \omega_{i}T_{g,i}$$

where ω_i_ is the mass fraction for each volatility bin, and here in this study, the fraction of signal intensity was utilized due to the lack of sensitivity information for all the organic compounds. As shown in Figure S9, the *T*_g_ of each individual chemical compound increases as their volatility decreases. Table S3 compares the *T*_g_ values from literature measurements and modeling of the two SOA investigated in this study. The *T*_g_ values derived from thermogram measurements fall within the range of previous studies, with differences ranging from 7 to 23 K depending on the precursors. The relatively accurate predictions of *T*_g_ validated the potential of VIA NH_4_^+^ Vocus in measuring the volatility distribution and deriving viscosity information with a higher time resolution.

The viscosity and characteristic diffusion time scale of SOA highly depend on the environmental RH.^62^ The viscosity and mixing time of water for 200 nm SOA particles as a function of RH were derived with the Gordon–Taylor equation and the Vogel–Tammann–Fulcher equation and are plotted in Figure 5. The shaded areas were constrained by applying two different *T*_g_ parametrizations (upper: Li et al., 2020,^76^ lower: Zhang et al., 2019)^80^ based on the same volatility distribution, providing a wider range of the derived viscosity under different environmental conditions. The effective hygroscopicity was set to be 0.1 ^111^ and 0.015 ^112^ for α-pinene and β-caryophyllene SOA, respectively. Compared with existing literature data of the α-pinene SOA, the viscosity derived using the VIA-CIMS is closely aligned with previous viscosity measurements.^113,114^ It is worth noting that a small underestimation of results from this work was observed when comparing with one previous study by Zhang et al., which could be partially explained by different experimental conditions, mass loadings, and O/C ratios.^115^ The mass loading in Zhang et al. was 70 μg/m^3^,^65^ which is lower than the 90 μg/m^3^ from this study. The higher mass loading would alter the gas-particle partitioning, and more volatile compounds would partition onto aerosol particles, leading to a lower viscosity estimation. Results also show that under the same RH condition, the viscosity of β-caryophyllene SOA was up to a few orders of magnitude higher than that of the α-pinene SOA. Similar to the viscosity, the equilibrium mixing time of α-pinene SOA changes drastically with RH from around 26 hr at dry conditions to around 5 ms at 80% RH.

**Figure 5:**
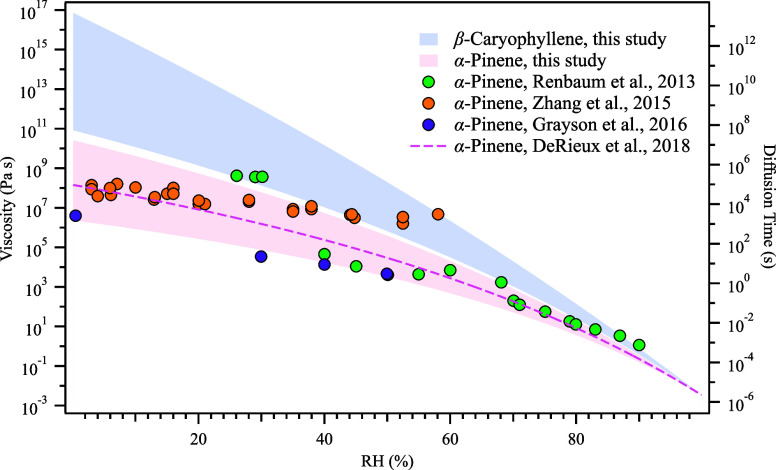
Viscosity and equilibrium mixing time of SOA as a function of RH. The two shaded areas represent the two SOA types generated in this study. The shaded ranges were determined with *T*_g_ calculated from different parametrizations (upper: Li et al., 2020,^76^ lower: Zhang et al., 2019).^80^ The dashed line and dots are from models^115^ and measurements^64,65,113,115^ of previous studies on α-pinene SOA, respectively.

However, the detected signals intensities may not directly correspond to individual mass loading because of unknown compound-specific sensitivities, leading to potential uncertainties in viscosity estimation. Given that multiple compounds were binned into a small number of volatility bins, the positive and negative biases in sensitivities may be canceled out or minimized compared to the individual bias from each compound. In addition, the thermal decomposition and unwanted reactions were cautiously reduced by lowering the residence time while maintaining efficient evaporation. Although only a small number of thermally decomposed compounds were detected in this study, thermal decomposition remains an important yet poorly constrained factor when analyzing highly thermally labile compounds, including certain organosulfates^116^ or acids.^117^

### 3.5 Atmospheric Implications

This study demonstrates a real-time technique for simultaneous quantification of chemical composition and volatility distribution using the VIA NH_4_^+^ Vocus CIMS, a novel thermal desorption unit coupled with a soft ionization CIMS. The volatility calibration from the thermal desorption temperature to the saturation mass concentration was conducted by atomizing the organic aerosol standard and evaporating with programmed temperature control. A series of organic compounds with a large range of logC* values from −4 to 3 can be successfully detected with the experimental setup. With NH_4_^+^·H_2_O as the reagent ion, the chemical formulas of particle-phase, highly oxygenated, SOA dimers were obtained. Investigating the thermograms for the detected organic compounds enables a comprehensive estimation of the volatility distribution of the SOA mixtures with different precursors, providing a useful tool to further refine the chemical models. The *T*_g_ derived from the online volatility measurements is shown to be accurate when compared with other methodologies, further improving understanding of the phase state of SOA. Future studies may be needed to examine the effects of residence time on VIA, as more thermal decomposition can happen with longer residence time.^81^ By providing near real-time quantification of volatility and viscosity, this study offers new insights into SOA evolution and advances our understanding of their physicochemical properties.

## Supplementary Material



## Notes

Time series of the VIA temperature ramping; aerosol volume and size distribution; comparison of volatility calibration with literature; calibration of evaporation enthalpy derived from this study and literature; mass defect plots for α-pinene and β-caryophyllene SOA; thermogram and fitting of clean total signals for α-pinene and β-caryophyllene SOA; thermogram of series of compounds for α-pinene and β-caryophyllene SOA; relation between volatility derived from evaporation enthalpy and *T*_50_; volatility basis set color-coded with derived *T*_g_; adopted saturation vapor pressure values for calibrants; chemical composition measured by AMS and VIA Vocus; and glass transition temperature derived from volatility (PDF)

## #

Osmo Laboratories, PBC, Somerville, Massachusetts, 02144, USA
