# Supplementary material for: Simultaneously Characterizing the Volatility Distribution and Phase State of Submicron Secondary Organic Aerosols Using a Vocus Vaporization Inlet for Aerosols with a Chemical Ionization Mass Spectrometer
Source: ACS EST Air. 2025 Oct 15;2(11):2410–21. doi: 10.1021/acsestair.5c00155 (PMC12624526; doi:10.1021/acsestair.5c00155)
Supplement: Supplementary file 1 [file ea5c00155_si_001.pdf]

SUPPORTING INFORMATION

Simultaneously Characterizing the Volatility Distribution and Phase State of Submicron Secondary Organic Aerosols (SOA) using a Vocus Vaporization Inlet for Aerosols with Chemical Ionization Mass Spectrometer (Vocus VIA-CIMS)

Sining Niu[1], Kyle P. McCary[1], Mitchell Alton[2], Jordan E. Krechmer[2, +], Harald Stark[2,3], Jason D. Surratt[4,5], Manjula Canagaratna[2], Yue Zhang[1]*

[1]*Department of Atmospheric Sciences, Texas A&M University, College Station, 77843, USA*

[2]*Center for Aerosol & Cloud Chemistry, Aerodyne Research, Billerica, 01821, USA*

[3]*Department of Chemistry, University of Colorado Boulder, and Cooperative Institute for Research in Environmental Sciences, Boulder, Colorado, 80309, USA*

[4]*Department of Environmental Sciences and Engineering, The University of North Carolina at Chapel Hill, Chapel Hill, North Carolina, 27599, USA*

[5]*Department of Chemistry, The University of North Carolina at Chapel Hill, Chapel Hill, North Carolina, 27599, USA*

[+]*Now at Osmo Labs, PBC, Somerville, MA, 02144, USA*

October 2025

[*]*Corresponding author:* Yue Zhang, yuezhang@tamu.edu

No. of pages: 17

No. of figures: 9

No. of tables: 4

S1. Sensitivity of Calibration Curve to PEG

To evaluate the influence of PEG on the volatility calibration curve, we performed a linear regression excluding PEG. The fitting result was $\log C^* = -17 + 6.5\times10^3\ T^{-1}$ and differed by less than 0.1 log C* from the original fit across the temperature range of interest. The calibration curve is robust and not overly influenced by PEG.

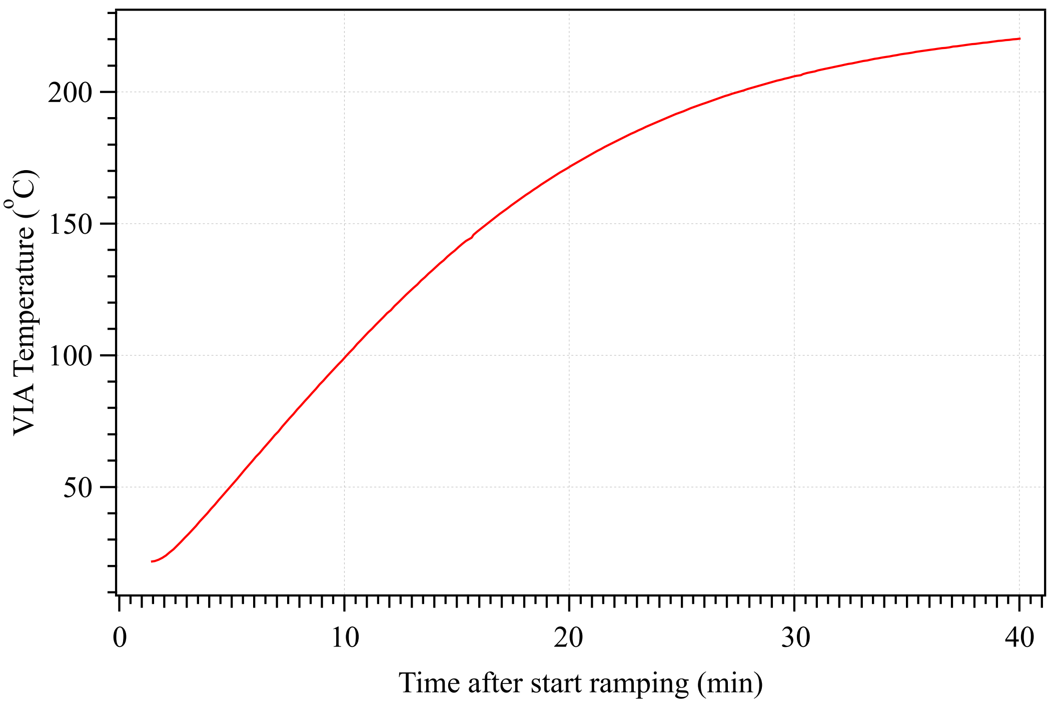

Figure S1. Time series of a typical ramping temperature profile of the VIA during experiments.

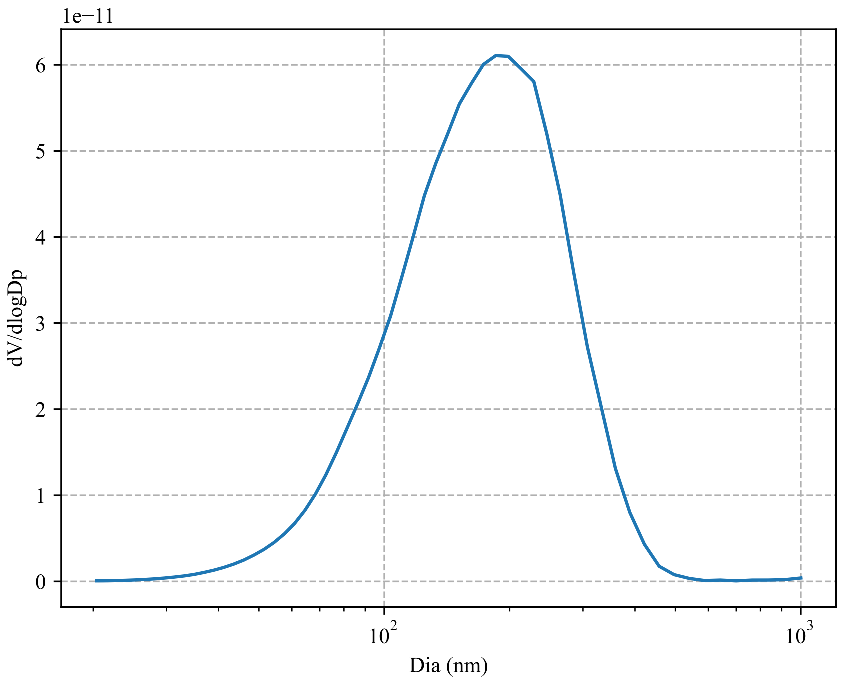

Figure S2. The volume and size distribution of self-nucleating particles during experiments**.**

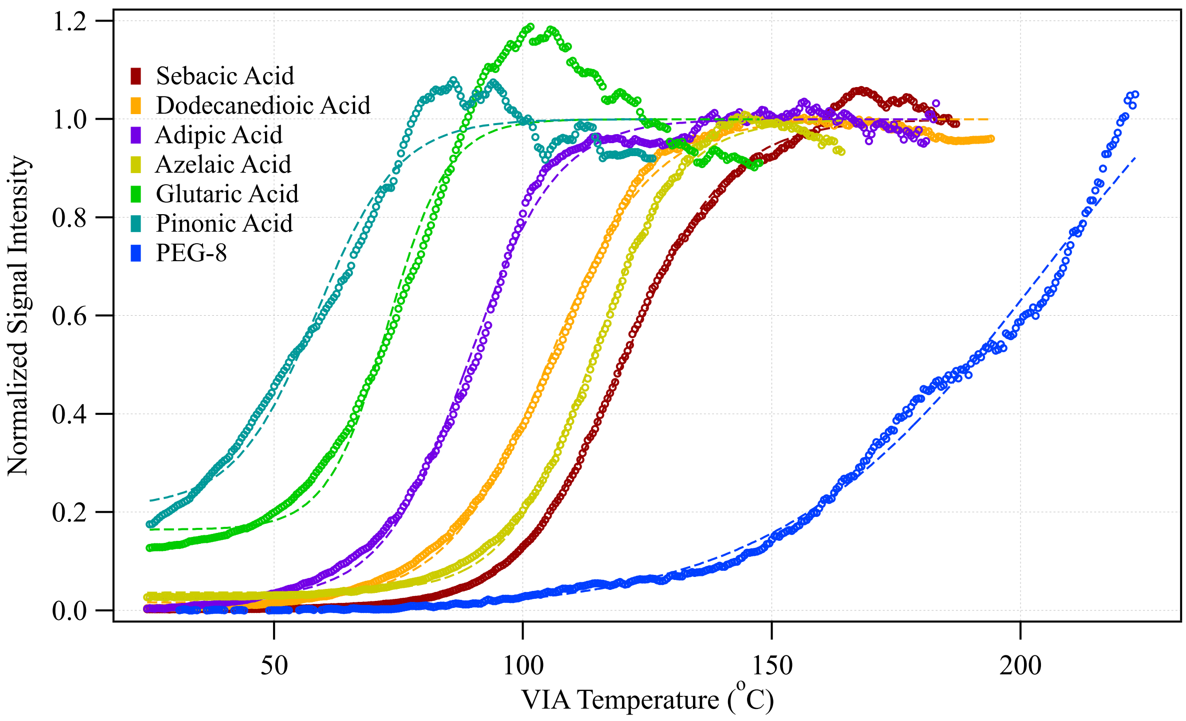

Figure S3. Thermogram for all the calibrants (circles) with their corresponding sigmoid fit (dashed lines).

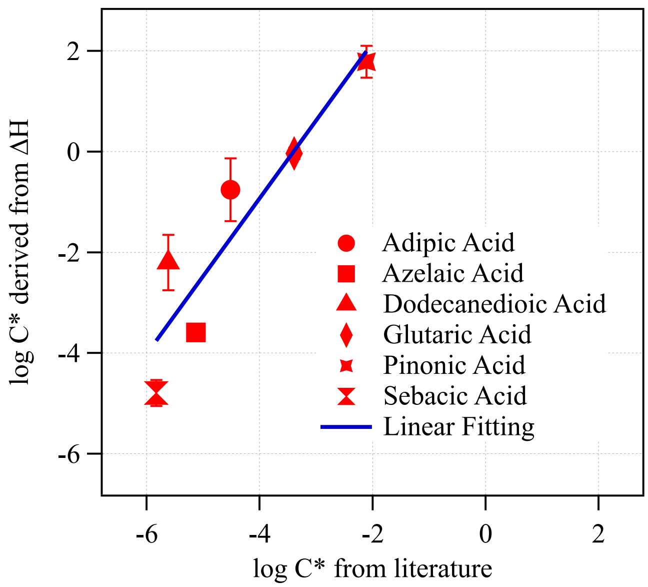

Figure S4. The logarithm of the saturation mass concentration C^* (in μg/m^3) for calibrants. The y-axis values are derived from the measured ΔH with the error bars denoting one standard deviation. The x-axis values are obtained from the literature. From the linear fit, logC^* from literature = (logC^* from delta H) × 0.65 − 3.4.

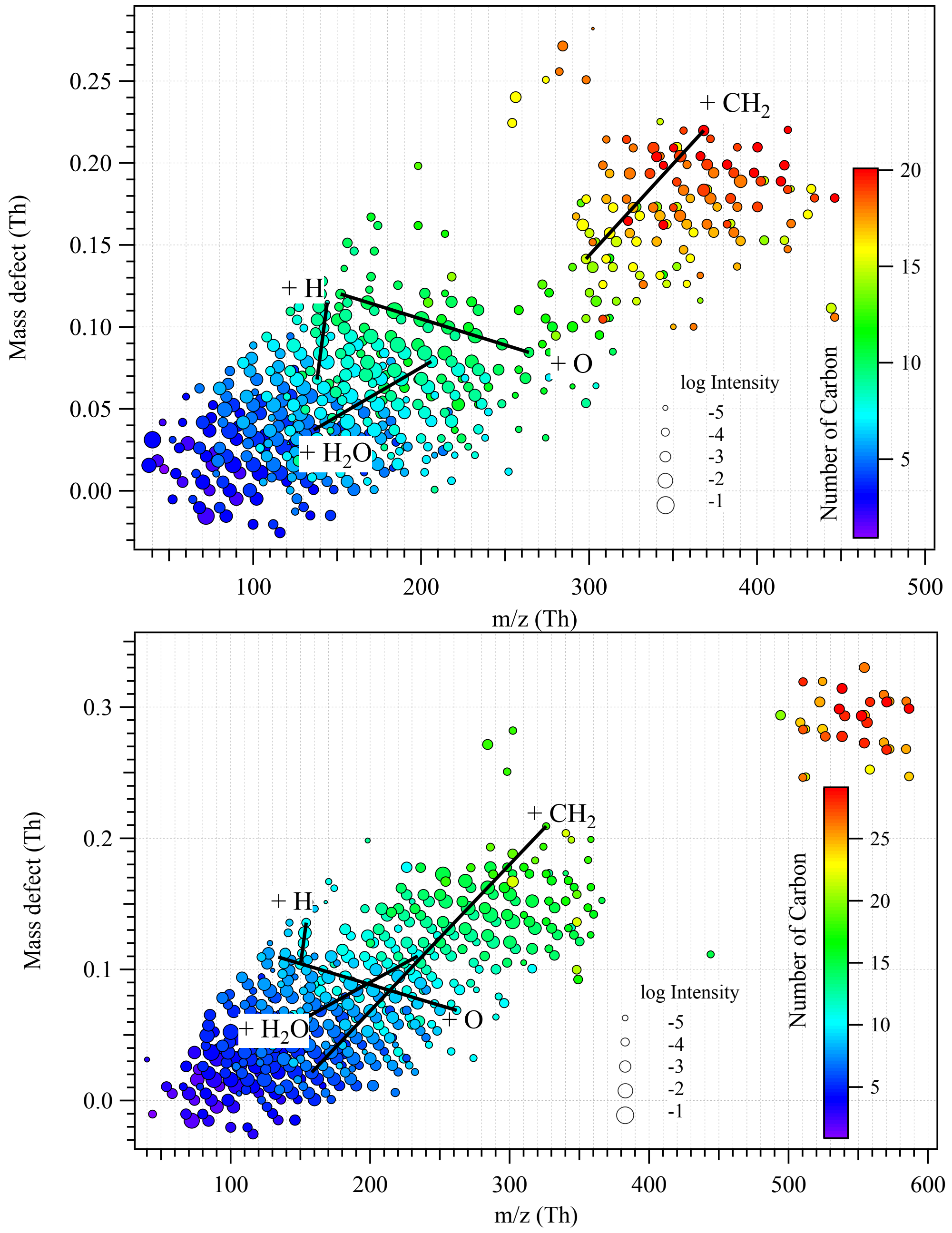

Figure S5. Mass defect plots of organic molecules identified from A) *α*-pinene and B) *β*-caryophyllene ozonolysis SOAs. The marker size is proportional to the logarithm of signal intensity (in cps). The linear trend lines indicate molecular changes due to the addition or removal of specific groups.

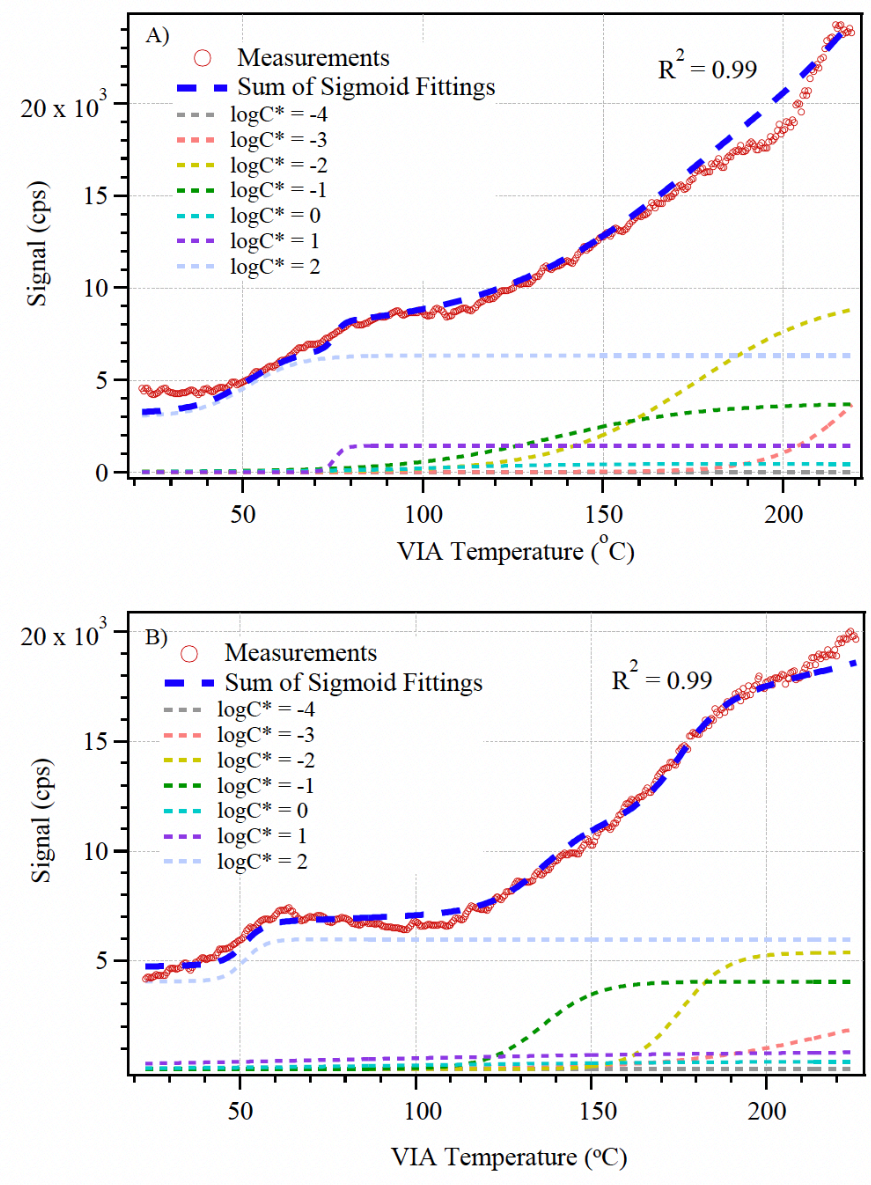

Figure S6. The thermogram and fit of processed total signals for A) *α*-pinene and B) *β*-caryophyllene SOA. The red circles represent data points from measurements, while the blue dashed lines represent multi-linear sigmoid fits. Other colored dashed lines represent sigmoid fits for each corresponding volatility bin.

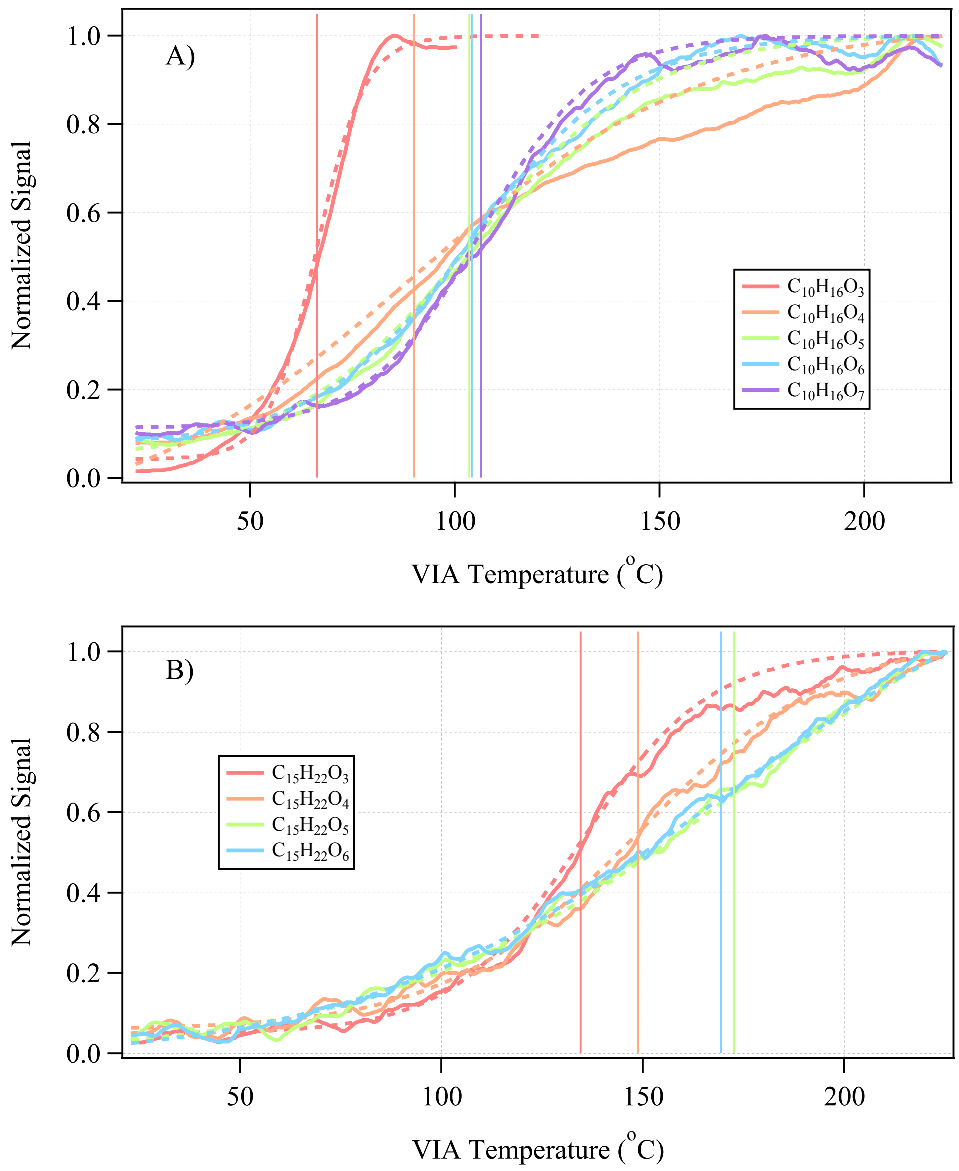

Figure S7. Thermogram of A) $C_{10}H_{16}O_x$ from *α*-pinene SOA and B) $C_{15}H_{22}O_x$ from *β*-caryophyllene SOA, with their respective T_{50} denoted by the vertical lines.

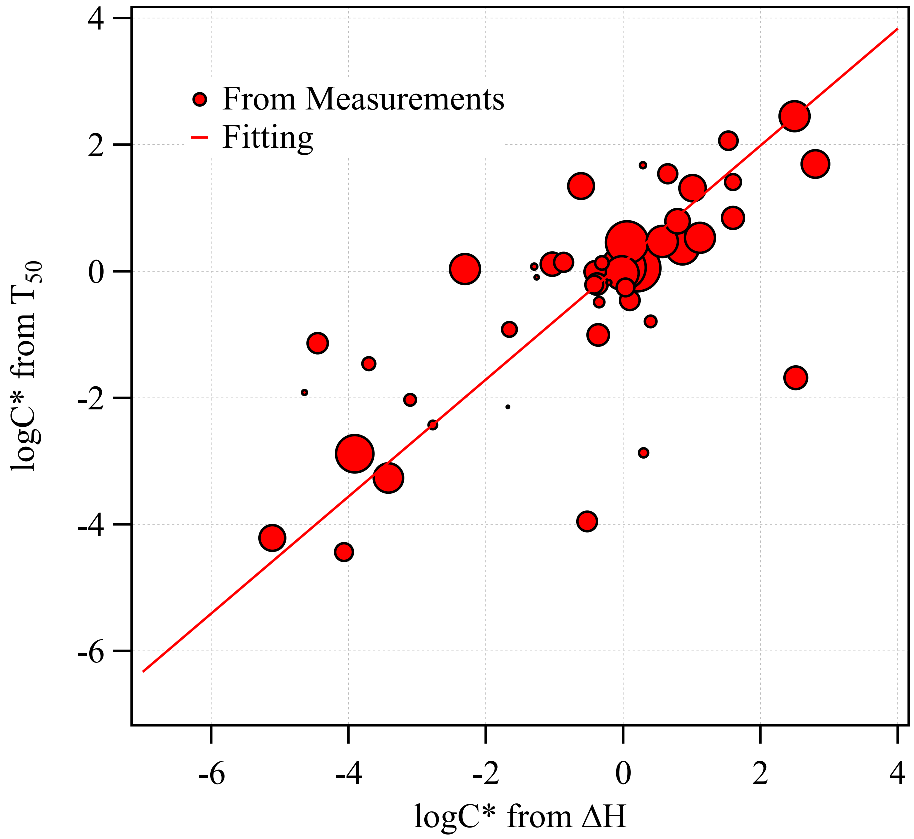

Figure S8. The logarithm of saturation mass concentration for SOA components. The x-axis is that derived from the measured Δ*H* after empirical correction with the linear relationship established in Figure S4. The y-axis is that derived from the T_{50} in thermogram. The marker size is proportional to the logarithm of signal intensity (in cps). The red line is the intensity weighted linear fit between the two log*C** values with a slope of 1.082 and an intercept of -0.152.

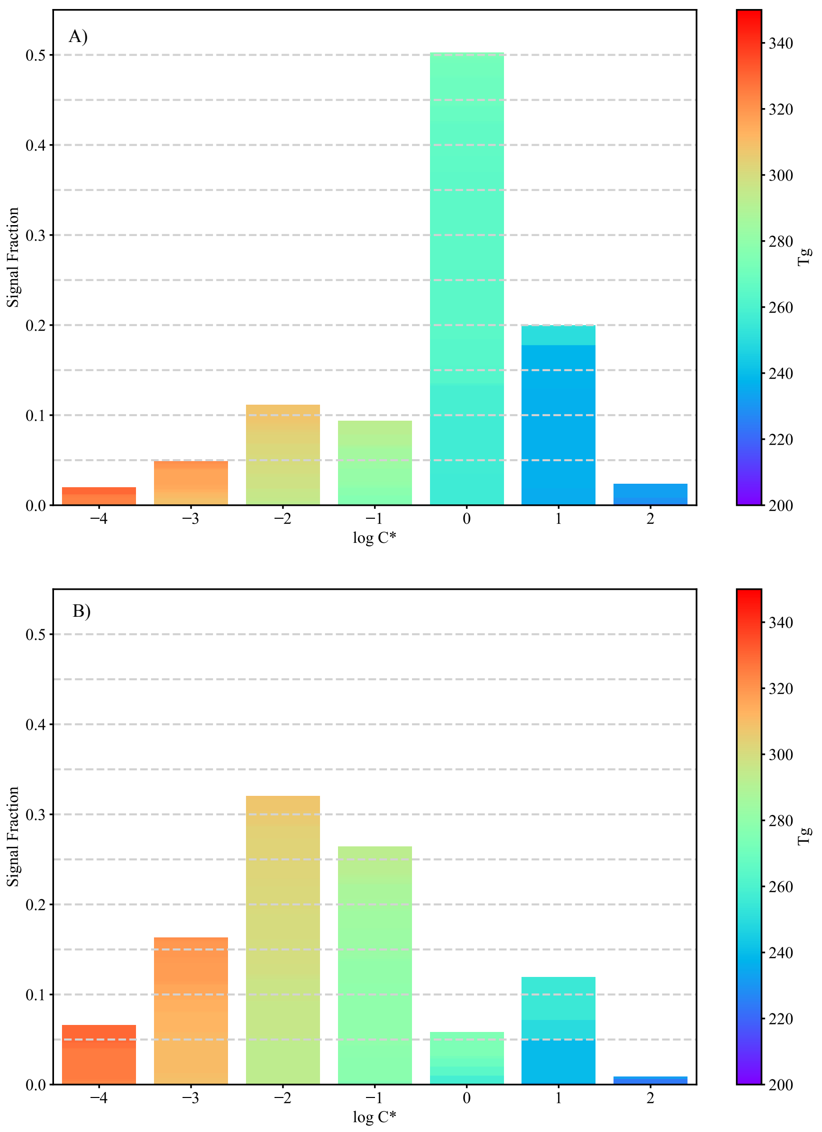

Figure S9. Volatility basis set color-coded with their glass transition temperature for A) α-pinene and B) β-caryophyllene SOA.

Table S1. Saturation vapor pressure values from literature of previous FIGAERO experiments and this study.

Saturation Pressure (Pa)	Lopez-Hilfiker et al., 2014[1]	Stark et al., 2017[2]	Nah et al., 2019[5]	Bannan et al., 2019[3]	This study
Glutaric acid		$10^{-3.17}$	$10^{-3.00}$	$10^{-3.00}$	$10^{-3.39}$
Cis-pinonic acid	$10^{-4.22}$	$10^{-1.52}$	$10^{-3.11}$	$10^{-3.11}$	$10^{-2.11}$
Azelaic acid	$10^{-5.22}$	$10^{-5.22}$	$10^{-5.13}$		$10^{-5.13}$
Sebacic acid	$10^{-5.82}$		$10^{-5.82}$		$10^{-5.83}$
Adipic acid				$10^{-3.74}$	$10^{-4.52}$
Dodecanedioic acid					$10^{-5.62}$
PEG-8					$10^{-7.04}$

Table S2. The O:C ratio, oxidation state, and average chemical composition derived from the AMS and Vocus VIA.

VOC Precursor	*α*-pinene	*β*-caryophyllene
O:C (AMS)	0.21	0.14
O:C (Vocus VIA)	0.56	0.58
OSc (AMS)	-0.93	-1.1
OSc (Vocus VIA)	-0.28	-0.30
Averaged Chemical Formula	$C_{6.1}H_{8.6}O_{3.4}$	$C_{6.7}H_{9.7}O_{3.8}$

Table S3. The glass transition temperatures derived from this study and previous studies.

Methodology	T_g of α-Pinene SOA (K)	T_g of β-Caryophyllene SOA (K)
Thermogram measurements (this study)	268	290
Dimer coagulation isolation and coalescence	272[8]	296[9]
Model simulation	275[10,11]-284[12]	267-277[13]

Table S4. Sensitivity analysis of thermal decomposition cutoff threshold for T_g and volatility distribution.

	T_g 10x cutoff (K)	T_g 5x cutoff (K)	T_g 100x cutoff (K)	VBS peak 10x cutoff (log C*)	VBS peak 5x cutoff (log C*)	VBS peak 100x cutoff (log C*)
α-Pinene SOA	268	268	268	0	0	0
β-Caryophyllene SOA	290	290	290	-2	-2	-2

References:

(1) Lopez-Hilfiker, F. D.; Mohr, C.; Ehn, M.; Rubach, F.; Kleist, E.; Wildt, J.; Mentel, T. F.; Lutz, A.; Hallquist, M.; Worsnop, D.; Thornton, J. A. A novel method for online analysis of gas and particle composition: description and evaluation of a Filter Inlet for Gases and AEROsols (FIGAERO). *Atmos. Meas. Tech.* **2014**, *7* (4), 983-1001. DOI: 10.5194/amt-7-983-2014.
(2) Stark, H.; Yatavelli, R. L. N.; Thompson, S. L.; Kang, H.; Krechmer, J. E.; Kimmel, J. R.; Palm, B. B.; Hu, W. W.; Hayes, P. L.; Day, D. A.; et al. Impact of Thermal Decomposition on Thermal Desorption Instruments: Advantage of Thermogram Analysis for Quantifying Volatility Distributions of Organic Species. *Environmental Science & Technology* **2017**, *51* (15), 8491-8500. DOI: 10.1021/acs.est.7b00160.
(3) Bannan, T. J.; Le Breton, M.; Priestley, M.; Worrall, S. D.; Bacak, A.; Marsden, N. A.; Mehra, A.; Hammes, J.; Hallquist, M.; Alfarra, M. R.; et al. A method for extracting calibrated volatility information from the FIGAERO-HR-ToF-CIMS and its experimental application. *Atmos. Meas. Tech.* **2019**, *12* (3), 1429-1439. DOI: 10.5194/amt-12-1429-2019.
(4) Joo, T.; Rivera-Rios, J. C.; Takeuchi, M.; Alvarado, M. J.; Ng, N. L. Secondary Organic Aerosol Formation from Reaction of 3-Methylfuran with Nitrate Radicals. *Acs Earth Space Chem* **2019**, *3* (6), 922-934. DOI: 10.1021/acsearthspacechem.9b00068.
(5) Nah, T.; Xu, L.; Osborne-Benthaus, K. A.; White, S. M.; France, S.; Ng, N. L. Mixing order of sulfate aerosols and isoprene epoxydiols affects secondary organic aerosol formation in chamber experiments. *Atmospheric Environment* **2019**, *217*, 116953. DOI: https://doi.org/10.1016/j.atmosenv.2019.116953.
(6) Ye, Q.; Wang, M.; Hofbauer, V.; Stolzenburg, D.; Chen, D.; Schervish, M.; Vogel, A.; Mauldin, R. L.; Baalbaki, R.; Brilke, S.; et al. Molecular Composition and Volatility of Nucleated Particles from α-Pinene Oxidation between −50 °C and +25 °C. *Environmental Science & Technology* **2019**, *53* (21), 12357-12365. DOI: 10.1021/acs.est.9b03265.
(7) Wang, M.; Chen, D.; Xiao, M.; Ye, Q.; Stolzenburg, D.; Hofbauer, V.; Ye, P.; Vogel, A. L.; Mauldin, R. L., III; Amorim, A.; et al. Photo-oxidation of Aromatic Hydrocarbons Produces Low-Volatility Organic Compounds. *Environmental Science & Technology* **2020**, *54* (13), 7911-7921. DOI: 10.1021/acs.est.0c02100.
(8) Petters, S. S.; Kreidenweis, S. M.; Grieshop, A. P.; Ziemann, P. J.; Petters, M. D. Temperature- and Humidity-Dependent Phase States of Secondary Organic Aerosols. *Geophys Res Lett* **2019**, *46* (2), 1005-1013. DOI: https://doi.org/10.1029/2018GL080563.
(9) Kasparoglu, S.; Perkins, R.; Ziemann, P. J.; DeMott, P. J.; Kreidenweis, S. M.; Finewax, Z.; Deming, B. L.; DeVault, M. P.; Petters, M. D. Experimental Determination of the Relationship Between Organic Aerosol Viscosity and Ice Nucleation at Upper Free Tropospheric Conditions. *Journal of Geophysical Research: Atmospheres* **2022**, *127* (16), e2021JD036296. DOI: https://doi.org/10.1029/2021JD036296.
(10) Bateman, A. P.; Bertram, A. K.; Martin, S. T. Hygroscopic Influence on the Semisolid-to-Liquid Transition of Secondary Organic Materials. *The Journal of Physical Chemistry A* **2015**, *119* (19), 4386-4395. DOI: 10.1021/jp508521c.
(11) Renbaum-Wolff, L.; Grayson, J. W.; Bateman, A. P.; Kuwata, M.; Sellier, M.; Murray, B. J.; Shilling, J. E.; Martin, S. T.; Bertram, A. K. Viscosity of α-pinene secondary organic material and implications for particle growth and reactivity. *Proceedings of the National Academy of Sciences* **2013**, *110* (20), 8014-8019. DOI: doi:10.1073/pnas.1219548110.
(12) Grayson, J. W.; Zhang, Y.; Mutzel, A.; Renbaum-Wolff, L.; Böge, O.; Kamal, S.; Herrmann, H.; Martin, S. T.; Bertram, A. K. Effect of varying experimental conditions on the viscosity of α-

pinene derived secondary organic material. *Atmos. Chem. Phys.* **2016**, *16* (10), 6027-6040. DOI: 10.5194/acp-16-6027-2016.
(13) Maclean, A. M.; Smith, N. R.; Li, Y.; Huang, Y.; Hettiyadura, A. P. S.; Crescenzo, G. V.; Shiraiwa, M.; Laskin, A.; Nizkorodov, S. A.; Bertram, A. K. Humidity-Dependent Viscosity of Secondary Organic Aerosol from Ozonolysis of β-Caryophyllene: Measurements, Predictions, and Implications. *Acs Earth Space Chem* **2021**, *5* (2), 305-318. DOI: 10.1021/acsearthspacechem.0c00296.
